# Screening of Plant Growth Regulators for Promoting Rooting of Pitaya Cuttings

**DOI:** 10.3390/plants15091357

**Published:** 2026-04-29

**Authors:** Chonghao Zhong, Chaofan Zheng, Meng Wang, Jiaying Sheng, Yikai Wang, Jiaquan Huang, Hua Tang, Yinhua Chen

**Affiliations:** 1 School of Tropical Agriculture and Forestry, Hainan University, Haikou 570228, China; 15105485077@163.com (C.Z.);13103028307@163.com (C.Z.); wm159357zdx@163.com (M.W.); 15240571981@163.com (J.S.); 15969664717@163.com (Y.W.); jqhuang@163.com (J.H.); 2 Institute of Sanya Breeding and Multiplication, Hainan University, Sanya 572025, China

**Keywords:** pitaya, cuttings, plant growth regulators, root growth, root morphology

## Abstract

Hainan is the dominant production area of the red-fleshed pitaya (*Hylocereus undatus*) cv. ‘Jindu No.1’ in China, and cutting propagation is the main method for its large-scale seedling cultivation. Plant growth regulators (PGRs) are the key factors regulating the rooting of cuttings. Existing studies mostly focus on the concentration optimization of a single agent, lack systematic broad-spectrum screening of commonly used PGRs in agriculture, and have the problem of disconnection between laboratory results and field production. To screen an efficient root-promoting PGR scheme suitable for large-scale seedling cultivation in Hainan production areas, this study established a three-level experimental system of “broad-spectrum primary screening→gradient re-screening→soil culture scenario verification”, used 14 kinds of PGRs commonly used in agricultural production as materials, and carried out a systematic evaluation combined with principal component analysis (PCA). 1-naphthaleneacetic acid (NAA), indole-3-acetic acid (IAA) and potassium indole-3-butyrate (K-IBA) were identified as high-efficiency agents in the primary screening, with a rooting rate of 100%, and the core root morphological indexes were significantly better than those of the water control (*p* < 0.05). Two independent experiments verified the stability of the “total growth–thickness” binary regulation mechanism of the pitaya root system. In the re-screening test, 400 mg·L^−1^ NAA had the best comprehensive performance, synergistically improving the total root growth and root thickness, and 125 mg·L^−1^ K-IBA had the most significant effect in promoting the longitudinal extension of roots, with the average root length increased by 760.0% compared with the control. Soil culture tests confirmed that the two optimal schemes had stable and reliable application effects in field substrate cultivation. The results of this study can provide technical support for the large-scale seedling cultivation of ‘Jindu No.1’ pitaya, and the established three-level screening system also provides a methodological reference for PGR screening in cutting propagation of similar tropical crops.

## 1. Introduction

Pitaya (*Hylocereus undatus*) is a perennial climbing plant belonging to the genus *Hylocereus* or *Selenicereus* of the Cactaceae family, which is native to the tropical and subtropical regions of the Americas [1,2]. Since its introduction to China in the 1990s, a large-scale industry has been developed in such provinces as Guangxi, Guangdong and Hainan. By 2023, the cultivated area of pitaya in China had exceeded 66,000 hectares (hm^2^) with an annual output of over 1.6 million metric tons (t), making China the world’s largest pitaya producer [3,4]. Hainan Province is located in the tropical monsoon climate zone, with abundant sunlight throughout the year and the coincidence of rainfall and high temperature in the same season [5,6], which makes it a core producing area for the red-fleshed pitaya ‘Jindu No.1’. This red-fleshed pitaya has been witnessing a steady rise in its cultivation area in Hainan in recent years due to its bright red peel, sweet flesh, stable commercial traits and remarkable economic benefits [7,8].

Plant growth regulators (PGRs) are a class of chemically synthetic or naturally extracted substances with phytohormone activity, which can regulate multiple key processes of plant growth and development [9,10,11,12]. Exogenous application of PGRs can alter the balance of endogenous hormones in cuttings and their redistribution among organs and activate cell division and differentiation, thereby inducing the formation of root primordia [13,14,15,16]. Meanwhile, PGRs can regulate the directional transport of nutrients such as carbohydrates and proteins, providing a material basis for the initiation and elongation of adventitious roots [17,18,19,20,21]. Selecting the appropriate type and concentration of PGR can significantly improve the rooting rate of cuttings, increase root number and root length, shorten the seedling raising cycle, and reduce production costs [22,23,24].

In commercial production, ‘Jindu No.1’ pitaya is mainly propagated by cuttings. Cutting propagation has the advantages of simple operation, rapid seedling formation and low cost, and can be used for annual batch seedling raising with little seasonal influence [25,26,27]. Studies have shown that PGRs are important external factors affecting the rooting of cuttings, which can significantly improve the rooting rate of cutting propagation [28,29,30]. The rooting speed and root development of pitaya cutting seedlings are also highly susceptible to the types and concentrations of PGRs, which further determine the seedling formation rate and subsequent growth potential [31,32,33,34,35]. Existing studies on PGR screening for pitaya cutting seedling mostly focus on the concentration gradient optimization of a single auxin agent, and lack systematic broad-spectrum screening and horizontal comparison of the 14 types of PGRs commonly used in agricultural production. Meanwhile, most studies only carry out effect evaluation based on laboratory culture systems, and lack verification of the stability of the screened schemes in the mainstream substrate cultivation mode of large-scale seedling raising, resulting in a disconnection between laboratory-optimized schemes and field-production requirements. To maximize the rooting rate, promote root development and screen for the optimal PGR formula, it is necessary to carry out a comparative study on the effects of different PGRs treatments on the root growth of cutting seedlings of this variety.

In this study, 14 kinds of PGR commonly used in agricultural production were selected, and a three-level system of “broad-spectrum primary screening→gradient re-screening→soil culture scenario verification” was established to systematically explore the regulatory effects of different types and concentrations of PGRs on the root growth of ‘Jindu No.1’ pitaya cuttings, and screen the optimal protocol for efficient root promotion. The high-efficiency protocols screened by the system were verified by pot experiments in production scenarios to ensure the field applicability of the screening results, and provide a theoretical basis and technical support for high-quality and large-scale seedling cultivation of pitaya.

## 2. Materials and Methods

### 2.1. Experimental Site and Plant Materials

This experiment was carried out in the Agricultural Science Base of Haidian Campus, Hainan University. During the experiment, the average temperature was about 26 °C, and natural light was used for illumination. The tested pitaya cuttings were the ‘Jindu No.1’ variety provided by the pitaya base of Hainan Enhong Agricultural Technology Co., Ltd., located in Baixue Village, Banqiao Town, Dongfang City, Hainan Province, China. Each cutting was about 20 cm in length. The 2–3 cm fleshy tissue at the base of the cutting was cut off to expose the xylem, and the cuttings were air-dried for 2–3 days until a waxy film formed on the wound surface prior to use.

### 2.2. Experimental Design

Both the primary screening (broad-spectrum screening) experiment and the re-screening (gradient optimization) experiment were conducted via hydroponic culture, while the soil culture verification experiment was carried out with substrate cultivation. The detailed experimental design, treatment settings and cultivation conditions for each stage are described in the following subsections.

All cuttings used in the experiments originated from the same batch of 3-year-old healthy *Hylocereus undatus* cv. ‘Jindu No.1’ mother plants with consistent growth status. For both the primary screening and re-screening tests, 10 cutting samples with no diseases or insect pests and with consistent growth status were selected for each treatment group, with 3 independent biological replicates. All cuttings were randomly assigned to each treatment group and randomly arranged in hydroponic tanks or soil pots to avoid positional effects caused by uneven light and temperature. The 2–3 cm base section of pitaya cuttings was subjected to regular root-dipping treatment daily, with a single root-dipping duration of 8–10 s. After the reagent was fully absorbed, the cuttings were placed back into the hydroponic tank for culture. The interval between single treatments was 24 h, the test cycle was 30 d, and the hydroponic nutrient solution was replaced every 3 d.

During the entire experimental period, daily visual inspection was performed to monitor the health status of all cuttings, including signs of stem rot, root browning and microbial contamination. The hydroponic nutrient solution was replaced every 3 days to inhibit microbial growth. No severe microbial contamination or root rot occurred in any treatment, and the total loss rate of cuttings due to rot or damage was less than 2% throughout the experiments.

The detailed information of all plant growth regulator (PGR) treatments across three experimental stages is summarized in Table 1.

#### 2.2.1. Primary Screening Experiment

The primary screening experiment was conducted from 16 March 2025 to 16 April 2025. The experiment was carried out via hydroponic culture, using a modified 1/2-strength Hoagland solution as the nutrient medium [36]. The specific formula of the nutrient solution is as follows: 1.75 mmol·L^−1^ (NH_4_)_2_SO_4_, 3.50 mmol·L^−1^ KNO_3_, 1.25 mmol·L^−1^ K_2_SO_4_, 2.50 mmol·L^−1^ CaCl_2_, 1.00 mmol·L^−1^ MgSO_4_·7H_2_O, 0.035 mmol·L^−1^ Fe-EDTA, 0.05 mmol·L^−1^ H_3_BO_3_, 0.01 mmol·L^−1^ MnSO_4_, 0.0008 mmol·L^−1^ ZnSO_4_·7H_2_O, 0.0002 mmol·L^−1^ CuSO_4_·5H_2_O, 0.0021 mmol·L^−1^ NaMoO_3_·2H_2_O, 0.0048 mmol·L^−1^ KI. A total of 15 treatment groups were set in this experiment, with clean water treatment as the blank control (CK). The types and mass concentrations of PGRs used in other treatments are shown in Table 1.

#### 2.2.2. Re-Screening Experiment

The re-screening experiment was conducted from 6 May 2025 to 6 June 2025. The hydroponic culture system and nutrient solution formula were completely consistent with those of the primary screening experiment (Section 2.2.1). A total of 16 treatment groups were set in this experiment, with clean water treatment as the blank control (CK). The types and mass concentrations of PGRs used in other treatments are shown in Table 1.

#### 2.2.3. Soil Culture Verification Experiment

Combined with the actual production operation habits of large-scale seedling cultivation, the pot experiment was carried out by substrate irrigation, which is the mainstream method in commercial production, to verify the application effect of the optimal protocols in actual seedling scenarios. The treatment concentration was consistent with the optimal concentration screened in the hydroponic test.

The soil culture verification test was conducted from 15 June 2025 to 15 July 2025. The mixed substrate of peat soil:perlite = 2:1 (volume ratio) was used in the test, and was disinfected with 0.5% potassium permanganate solution, then filled into seedling nutrition pots, and watered thoroughly with bottom water for later use. A total of 3 treatment groups were set up in the test, with clean water treatment as the blank control (CK). The types and mass concentrations of PGRs used in other treatments are shown in Table 1. All treatments were carried out by substrate irrigation, which was performed once at 0 d, 7 d and 14 d after the start of the test, respectively. Each plant was irrigated with 100 mL of the corresponding reagent solution each time to ensure that the reagent evenly penetrated into the root distribution area. The test cycle was 30 d, during which the substrate was kept moist, and no other fertilizers or reagents were applied.

The sources and specifications of all PGR reagents used in the experiment are detailed in Table 2.

Preparation of PGR working solutions: All PGR reagents except indole-3-acetic acid (IAA) were directly prepared into working solutions with deionized water. Since IAA is slightly soluble in water, a small amount of anhydrous ethanol was used to fully dissolve IAA first, and then the solution was diluted to the target concentration with a large volume of deionized water. The final volume fraction of ethanol in all IAA working solutions was less than 0.1%, and its potential effect on the rooting of pitaya cuttings was negligible.

### 2.3. Determination of Indicators and Methods

Data collection for root morphological traits was performed in a single-blind manner. The measurer was unaware of the treatment grouping information to avoid subjective measurement bias.

#### 2.3.1. Dynamic Observation of Rooting

The occurrence time of adventitious roots of cuttings was observed daily, and the number of days when the first adventitious root broke through the epidermis was recorded. At the end of the test, the rooting rate was counted, and the calculation formula was:

Rooting rate (%) = (Number of rooted cuttings/Total number of cuttings) × 100%

#### 2.3.2. Determination of Root Morphological Traits

At the 30th day of the test, the whole plant was taken out with the root system kept intact and was rinsed with deionized water, and the external water was blotted with absorbent paper. Roots ≥ 1 cm were regarded as effective roots.

All average root morphological indicators, including average root number, average root length, average root surface area, average root diameter, and average root volume, were calculated as the ratio of the total corresponding value to the number of rooted cuttings.

Root morphological traits were measured by root scanning analysis system (WinRHIZO Pro 2020, Regent Instruments Inc., Quebec, QC, Canada).

### 2.4. Data Statistics and Analysis

The experimental data were sorted and calculated by Microsoft Excel 2021(Microsoft Corp., Redmond, WA, USA). The replicate unit of the experiment was the cuttings in a single pot, with 10 plants per treatment and 3 independent biological replicates. The average value of the 3 replicates was used as the final measurement result of the indexes.

Statistical analysis was performed using SPSS 27.0 software (IBM Corp., Armonk, NY, USA). One-way analysis of variance (ANOVA) was used for the primary screening, re-screening and soil culture verification tests, and Duncan’s new multiple range test was used for multiple comparisons, with the significance level set at α = 0.05 (*p* < 0.05).

The raw data were standardized by Z-score prior to principal component analysis (PCA). Principal components were extracted according to the criteria of eigenvalue > 1 and cumulative explained variance ≥ 85%. The weight of each principal component was calculated based on its explained variance, and the comprehensive score was obtained accordingly.

Graphs were drawn by Origin 2024 software (OriginLab Corp., Northampton, MA, USA); images were optimized by Photoshop 2024 (Adobe Systems Inc., San Jose, CA, USA).

## 3. Results and Analysis

### 3.1. Analysis of Primary Screening Results

#### 3.1.1. Effects of Different PGR Treatments on Rooting Time and Rooting Rate of Pitaya

**Figure 1:**
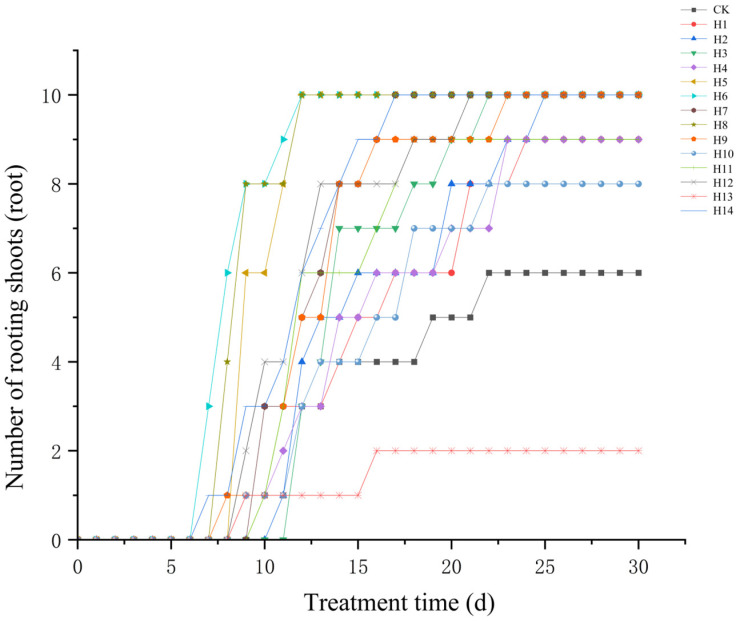
Variation trend of the number of rooted stem cuttings under different PGR treatments over time.

As shown in Figure 1, the earliest rooting time (Tfirst) and time to reach maximum root number (Tmax) of the tested cuttings differed significantly among treatments. The first adventitious root appeared in H6 treatment on the 7th day of the test, which was 3 d earlier than the CK treatment; followed by H5 and H4 treatments, where adventitious roots appeared on the 9th day of the test, 1 d earlier than the CK treatment. H5, H6, H8 and H14 treatments reached the maximum root number within 12–17 d, while the CK treatment took 22 d. This indicated that appropriate PGRs could advance the occurrence of adventitious roots and significantly shorten the rooting cycle.

**Figure 2:**
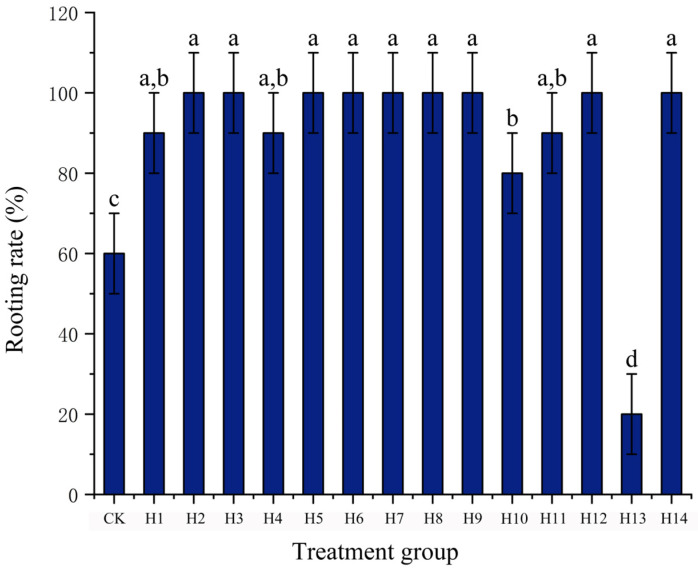
Effects of different PGR treatments on rooting rate. Different lowercase letters above the bars indicate significant differences among treatments at *p* < 0.05 according to Duncan’s new multiple range test.

As shown in Figure 2, the rooting rate of each treatment ranged from 20% to 100% measured at 30 d of the test. Among them, 9 treatments, including H2, H3, H5–H9, H12 and H14 reached a rooting rate of 100%, which was significantly higher than that of the CK treatment (*p* < 0.05); H1, H4, H11 (90%) and H10 (80%) treatments were significantly higher than the CK treatment (*p* < 0.05); the rooting rate of the H13 treatment was only 20%, which was significantly lower than other treatments (*p* < 0.05, Duncan’s new multiple range test).

Taking rooting rate ≥ 90% and Tmax ≤ 20 d as the criteria, 10 treatments including H2, H3, H5–H9, H12 and H14 met the requirements of rapid rooting and high survival rate. Among them, H5, H6, H8 and H14 treatments had the advantages of the earliest rooting (≤9 d) and the fastest reaching peak root number (≤12 d).

#### 3.1.2. Effects of Different PGR Treatments on Root System of Pitaya

**Figure 3:**
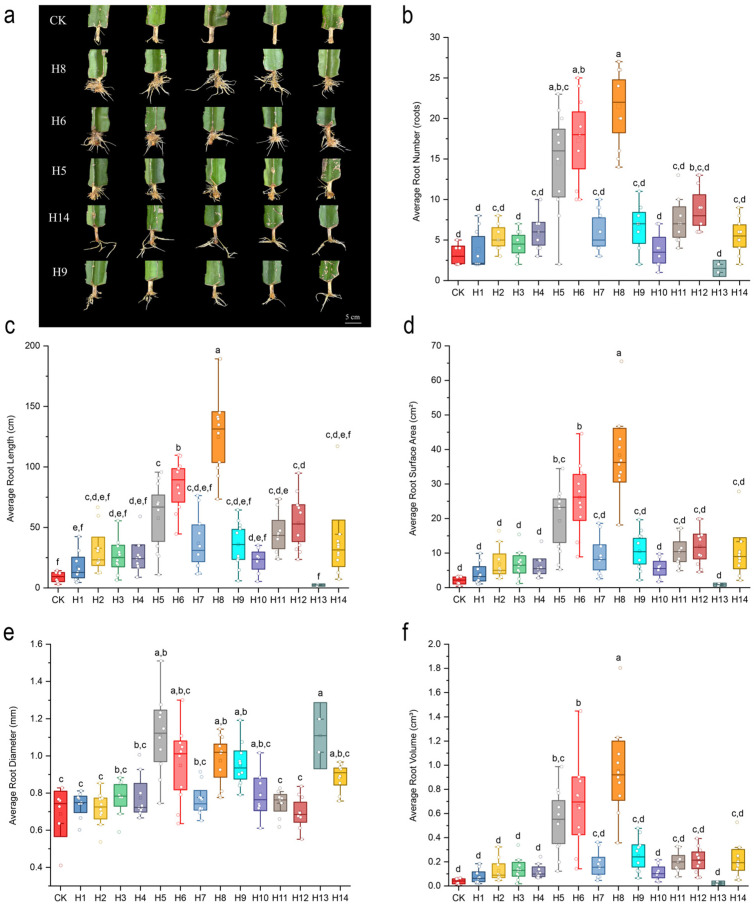
Root phenotype and morphological indices of pitaya cuttings under different PGR treatments in the primary screening experiment. (**a**) Root phenotype of pitaya cuttings under different treatments (top 5 treatments); (**b**) average root number; (**c**) average root length; (**d**) average root surface area; (**e**) average root diameter; (**f**) average root volume. Different lowercase letters indicate significant differences among treatments at *p* < 0.05 by Duncan’s new multiple range test.

The rooting phenotype and quantitative root morphological traits of pitaya under different PGR treatments are shown in Figure 3.

Overall, all PGR treatments promoted the rooting of pitaya cuttings to varying degrees. H8 and H6 treatments showed the most vigorous root growth, while the CK group had the weakest rooting performance with the fewest and shortest roots (Figure 3a).

As shown in Figure 3b–f, H8, H6 and H5 treatments significantly improved all root morphological indexes of pitaya cuttings compared with the CK group (*p* < 0.05), among which H8 treatment showed the best overall rooting-promoting effect, with the highest average root number, root length, root surface area and root volume among all treatments. H5 treatment exhibited the most significant effect on promoting root radial thickening, with the largest average root diameter. In contrast, H13 treatment (20 mg·L^−1^ CPPU) showed an obvious inhibitory effect on root growth, with no significant difference in most root indexes compared with the CK group (*p* > 0.05). Detailed raw data of root morphological indicators for all treatments in the primary screening experiment are listed in Table S1 (Supplementary Materials).

#### 3.1.3. Principal Component Analysis and Comprehensive Evaluation of Different PGR Treatments Based on Root Morphological Indicators

The comprehensive score of each treatment was calculated as: F = PC_1_ × W_1_ + PC_2_ × W_2_, where F represents the comprehensive score, PC_1_ and PC_2_ represent the scores of the two principal components, and W_1_ and W_2_ represent their corresponding variance contribution weights.

**Figure 4:**
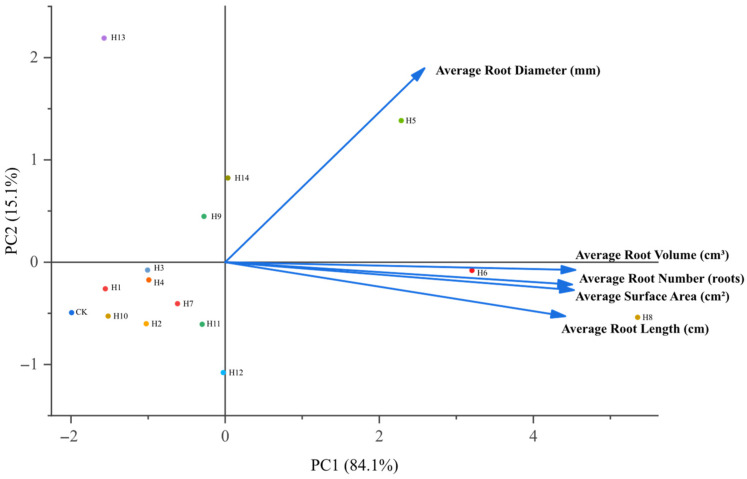
Biplot of principal component analysis of root morphological indicators in the primary screening experiment. PC1 explained 84.07% of the total variance, and PC2 explained 15.17% of the total variance.

Principal component analysis (PCA) was performed to comprehensively evaluate the rooting performance of pitaya cuttings under different PGR treatments, and the results are presented in Figure 4. The first two principal components accounted for 99.24% of the total variance and represented most of the information from the original indicators. Root length, root surface area and root volume showed strong positive loadings on PC1, while root diameter exhibited a negative loading on PC1. The PCA biplot clearly distinguished different treatments, among which H8 and H6 were clustered together and distinctly separated from the CK group, indicating that these two treatments significantly improved the comprehensive rooting performance of pitaya cuttings.

Detailed PCA results, including eigenvalue decomposition and factor loading matrix, and comprehensive scores of all treatments are provided in Tables S2–S4 (Supplementary Materials).

### 3.2. Analysis of Re-Screening Results

#### 3.2.1. Effects of PGR Treatments with Different Concentrations on Root System of Pitaya

**Figure 5:**
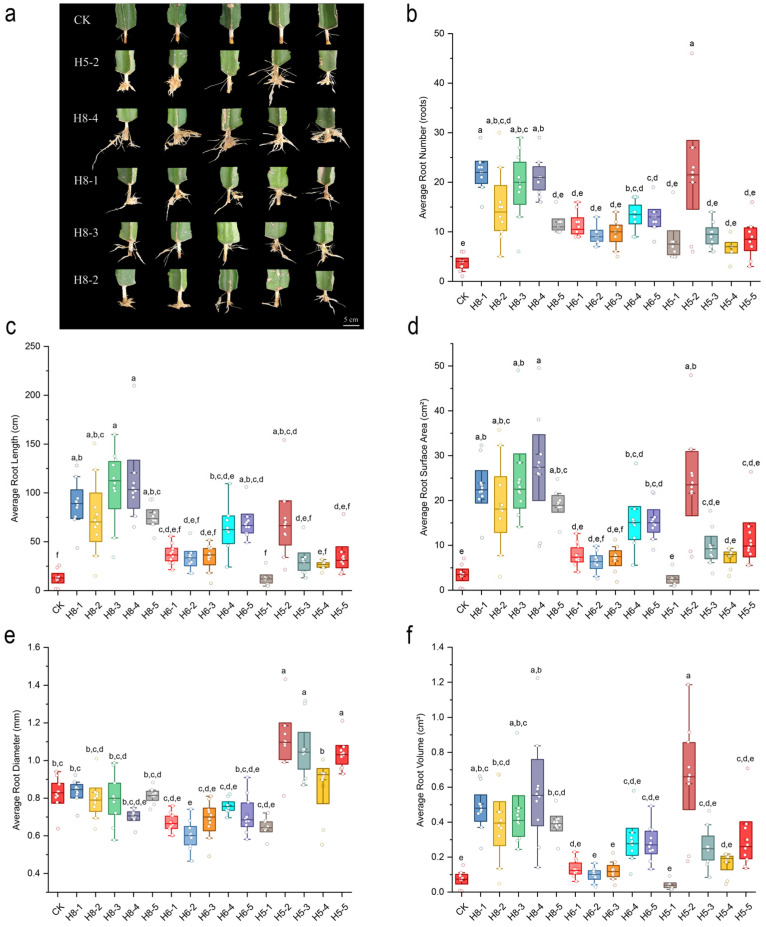
Root phenotype and morphological indices of pitaya cuttings under different PGR concentrations in the re-screening experiment. (**a**) Root phenotype of pitaya cuttings under different treatments (top 5 treatments); (**b**) average root number; (**c**) average root length; (**d**) average root surface area; (**e**) average root diameter; (**f**) average root volume. Different lowercase letters indicate significant differences among treatments at *p* < 0.05 by Duncan’s new multiple range test.

The rooting phenotype and quantitative root morphological traits of pitaya under different PGR concentrations in the re-screening experiment are shown in Figure 5.

Overall, all PGR treatments promoted the rooting of pitaya cuttings to varying degrees. H5-2 and H8-4 treatments showed the most vigorous root growth, while the CK group had the weakest rooting performance with the fewest and shortest roots (Figure 5a).

As shown in Figure 5b–f, the K-IBA (H8) series showed the most stable promoting effect on total root growth, among which H8-4 treatment had the highest average root length, root surface area and root volume. The NAA (H5) series showed extreme concentration sensitivity, with H5-2 treatment achieving the best comprehensive performance. This was the only treatment that realized synergistic improvement of total root growth and radial thickening, with the largest average root diameter and root volume. The IAA (H6) series showed weak rooting-promoting effects, with no significant difference in most root indexes compared with the CK group at high concentrations (*p* > 0.05). Detailed raw data of root morphological indicators for all concentration gradient treatments in the re-screening experiment are listed in Table S5 (Supplementary Materials).

#### 3.2.2. Principal Component Analysis and Comprehensive Evaluation of PGR Treatments with Different Concentrations Based on Root Morphological Indicators

The comprehensive score of each treatment was calculated using the same formula as the primary screening experiment.

**Figure 6:**
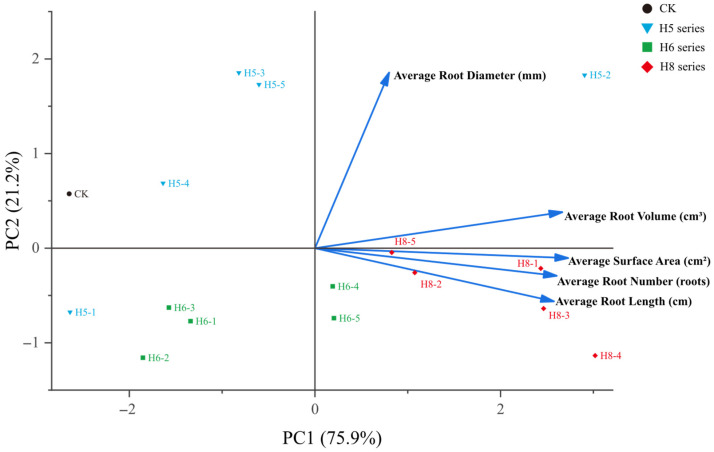
Biplot of principal component analysis of root morphological indicators in the re-screening experiment. PC1 explained 75.85% of the total variance, and PC2 explained 21.18% of the total variance.

Principal component analysis (PCA) was employed to comprehensively assess the rooting performance of pitaya cuttings under different concentrations of PGRs, with the results illustrated in Figure 6. The first two principal components explained 97.03% of the total variation, which could adequately reflect the original information of all root indices. Root number, root length and root volume exerted strong positive loadings on PC1, whereas root diameter showed a negative loading on PC1.

The three compound series exhibited distinct concentration–response patterns in the biplot: the NAA (H5) series showed extreme concentration sensitivity, with the optimal treatment H5-2 clearly separated from all other NAA gradients; the K-IBA (H8) series showed stable rooting-promoting effects across medium and low concentrations, with multiple gradients clustered in the positive direction of PC1; while the IAA (H6) series was concentrated near the coordinate origin, with no significant separation from the CK group.

H8-4 and H5-2 were closely grouped and distinctly distinguished from the CK group, demonstrating that these two treatments significantly enhanced the overall rooting capacity of pitaya cuttings.

Detailed data regarding eigenvalue decomposition, factor loading matrix and comprehensive scores of all treatments are summarized in Tables S6–S8 (Supplementary Materials).

### 3.3. Analysis of Soil Culture Verification Results

**Figure 7:**
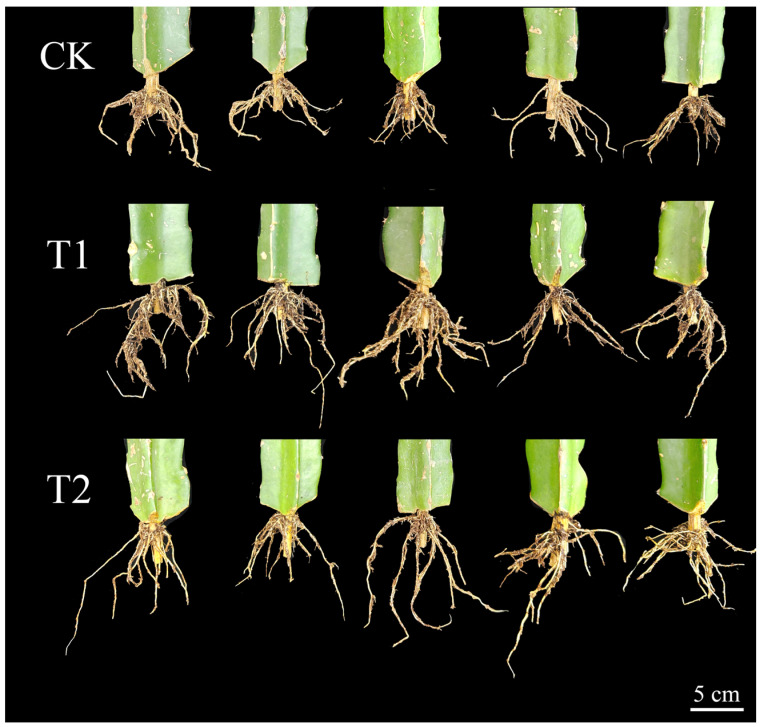
Root phenotype of pitaya under different treatments in the soil culture verification test.

Based on the comprehensive ranking and regulatory characteristics of the re-screening test, H5-2 (400 mg·L^−1^ NAA) ranked first in the comprehensive score, and was the only treatment that achieved synergistic improvement of total root growth and radial thickening; H8-4 (125 mg·L^−1^ K-IBA) ranked second, with cross-round stable advantages in longitudinal root extension, fibrous root differentiation and total root biomass accumulation. The two treatments represented two distinct root regulatory modes suitable for different production scenarios, and were the optimal concentration gradients of the corresponding agents screened by the two rounds of independent experiments. Therefore, the above two optimal treatments were selected for soil culture substrate seedling verification, to confirm their application stability and regulatory consistency in the actual commercial seedling production scenarios. The root phenotype of pitaya cuttings under different treatments in the soil culture verification test is shown in Figure 7.

The results of Table 3 showed that both T1 and T2 treatments could significantly improve the root morphological traits of pitaya cuttings (*p* < 0.05). T1 treatment continued the regulatory characteristics of K-IBA in hydroponics, with outstanding advantages in total root growth. The average root number was 26.4, the average root length was 351.11 cm, and the average root surface area was 71.37 cm^2^, which were significantly increased by 83.3%, 63.5% and 58.6%, respectively, compared with CK treatment, and were the best among all treatments. T2 treatment maintained the regulatory advantages of NAA in hydroponics, with the best performance in radial root development. The average root diameter was 0.85 mm and the average root volume was 1.26 cm^3^, which were significantly increased by 29.2% and 67.1%, respectively, compared with CK treatment (*p* < 0.05).

There was no significant difference in the average root diameter between T1 treatment and CK treatment (*p* > 0.05), but the root volume was increased by 53.8% compared with CK treatment. The average root number, length and surface area of T2 treatment were significantly higher than those of CK treatment (*p* < 0.05), with a stable overall root-promoting effect. The regulation laws of soil culture and hydroponic tests were highly consistent, indicating that the two optimal schemes screened in this study were not affected by cultivation methods, and had high reliability for large-scale production and popularization.

## 4. Discussion

This study aimed to address two key limitations in pitaya cutting seedling production: the lack of systematic broad-spectrum screening of commonly used plant growth regulators (PGRs), and the disconnection between laboratory screening results and field production requirements. Through a three-level experimental system of “broad-spectrum primary screening→gradient re-screening→soil culture scenario verification”, we identified two stable and efficient root-promoting protocols for ‘Jindu No.1’ pitaya cuttings: 400 mg·L^−1^ 1-naphthaleneacetic acid (NAA) and 125 mg·L^−1^ potassium indole-3-butyrate (K-IBA). We further identified a stable binary regulatory pattern of pitaya root morphology: “total growth–radial thickening”, which provides both technical support for commercial pitaya seedling cultivation and a methodological reference for PGRs screening in cutting propagation of tropical horticultural crops.

The two independent rounds of primary and re-screening experiments showed high consistency in the regulatory effects of PGRs on pitaya root morphology, confirming the reliability of our screening results. Principal component analysis (PCA) of both rounds revealed a stable binary regulatory mechanism of the pitaya root system: root number, root length, root surface area and root volume were highly synergistic and constituted the core dimension of “total root growth”, while root diameter was an independent regulatory dimension of “radial thickening”. This binary pattern revealed by PCA may be attributed to the distinct cellular and hormonal regulatory pathways underlying different root morphological traits: the “total root growth” dimension is likely driven by auxin-induced periclinal division of root primordium cells and longitudinal elongation of root epidermal cells, which co-determine root number, length, surface area and volume [37]; the “radial thickening” dimension is likely regulated by auxin-mediated anticlinal division of vascular cambium cells and secondary xylem differentiation, which specifically governs root diameter independently of longitudinal root growth [38], and the functional independence of these two cellular processes may explain their stable separation in the PCA loading matrix. This two-dimensional regulatory framework was consistent with the root morphological regulation pattern found in woody fruit crops such as apple and grape [39,40], indicating that it may be a common regulatory law for adventitious root formation in fruit crop cuttings, and can be used as a standardized evaluation system for the cutting rooting quality of tropical horticultural crops.

Auxin regulators were the only PGRs that achieved 100% rooting rate and significant promotion of root growth in both rounds of experiments, while non-auxin regulators showed only moderate or even inhibitory effects on pitaya cutting rooting. Among them, NAA and K-IBA exhibited stable and efficient root-promoting effects across two independent experiments, while IAA showed high fluctuation in regulatory efficiency, which was consistent with the performance of these auxins in cutting propagation of other succulent fruit crops [13].

The distinct root architectures induced by NAA and K-IBA are derived from their different metabolic characteristics and cellular regulatory modes. As a synthetic auxin resistant to enzymatic degradation in plants, NAA may be stably accumulated in the basal tissue of cuttings for a long time. It may preferentially promote the periclinal division of vascular cambium cells and the differentiation of secondary xylem, which not only drives the longitudinal elongation of adventitious roots, but also significantly promotes radial thickening of the root system [41,42,43]. This synergistic regulation of total growth and radial thickening may explain why 400 mg·L^−1^ NAA treatment achieved the highest comprehensive score in the re-screening experiment. In contrast, K-IBA, as the water-soluble potassium salt of IBA, is thought to be gradually converted into active indole-3-acetic acid through β-oxidation in plant cells to exert its auxin activity [44,45]. This slow conversion mode may avoid the inhibitory effect of high-concentration auxin on root meristem, and continuously promote the initiation of lateral root primordia and the elongation of fibrous roots, thus likely inducing a well-branched root system with large total length and absorption area [46].

The unstable regulatory effect of IAA may be mainly attributed to its inherent metabolic characteristics. As a natural auxin, IAA is likely rapidly degraded by endogenous indoleacetic acid oxidase in pitaya stem tissues, which may result in an extremely short effective action period under the rapid root-dipping treatment mode adopted in this study [47]. This may also explain why IAA showed a moderate root-promoting effect in the primary screening, but failed to maintain high efficiency in the re-screening experiment with the same treatment mode. For commercial large-scale production, the application effect of IAA may be stabilized by prolonged soaking treatment, split application, or adding antioxidant stabilizers to inhibit its enzymatic degradation. In addition, 20 mg·L^−1^ CPPU treatment showed a significant inhibitory effect on pitaya cutting rooting. This observation may be attributed to the well-documented mechanism that high concentrations of cytokinin can antagonize auxin-induced adventitious root differentiation [48,49], suggesting that cytokinin regulators should be used with caution during the rooting stage of pitaya cuttings.

The two optimal protocols (400 mg·L^−1^ NAA and 125 mg·L^−1^ K-IBA) maintained completely consistent regulatory characteristics in both hydroponic and soil culture systems: NAA still dominated radial thickening of the root system, while K-IBA still showed outstanding advantages in promoting total root length and fibrous root branching. This cross-cultivation-system consistency confirmed that the two protocols have stable application effects in the mainstream substrate cultivation mode of commercial pitaya seedling. Notably, the absolute values of all root morphological indexes in the soil culture test were significantly higher than those in the hydroponic test. Three potential mechanisms may contribute to this difference: first, the peat–perlite mixed substrate may provide a more stable water–air ratio and rhizosphere oxygen environment, which may be more conducive to the continuous growth of adventitious roots than hydroponic culture; second, the substrate may harbor beneficial rhizosphere microorganisms that can synergize with exogenous auxins to promote root growth; third, the substrate irrigation mode adopted in the soil culture test may achieve a longer effective exposure time of the cutting base to PGRs than the short-term root-dipping mode in hydroponics, thus potentially enhancing the root-promoting effect.

The two optimal protocols screened in this study have clear targeted application scenarios in commercial pitaya seedling production. The 125 mg·L^−1^ K-IBA treatment is most suitable for plug-tray rapid seedling cultivation: it can induce a well-developed fibrous root system with large absorption area, which can significantly improve the survival rate of transplanted seedlings and shorten the seedling slowing period after transplanting. The 400 mg·L^−1^ NAA treatment is more suitable for direct field cutting propagation: it promotes significant radial thickening of the root system, which can achieve rapid root fixation of cuttings in the field, enhance the drought resistance and wind resistance of seedlings, and reduce the management cost of field seedling cultivation. Compared with previous studies on pitaya cutting propagation, this study has two core innovations: first, we established a three-level screening system that links laboratory screening to field production, which effectively avoids the disconnection between laboratory optimized schemes and actual production requirements; second, we revealed the binary regulatory mechanism of pitaya root morphology, which provides a standardized evaluation system for rooting quality of pitaya cuttings, rather than only taking rooting rate as the single evaluation index.

This study has several limitations that need to be addressed in future research. First, the study only focused on the 30-day short-term rooting effect of PGRs, and did not track the long-term effects of the two optimal protocols on the transplanting survival rate, vegetative growth and fruit yield of pitaya seedlings in the field. Second, we did not conduct quantitative analysis of endogenous hormone dynamics in the cutting base during adventitious root formation, which limits the in-depth interpretation of the molecular regulatory mechanism of exogenous PGRs. In follow-up research, we will carry out long-term field plot experiments to verify the effects of the two optimal root-promoting protocols on the seedling formation rate, early growth and subsequent fruit yield of pitaya. Meanwhile, we will quantitatively analyze the dynamic changes in endogenous hormones during adventitious root formation, to further reveal the molecular mechanism of NAA and K-IBA regulating pitaya root morphogenesis. In addition, we will explore the synergistic effect of PGRs and beneficial rhizosphere microorganisms on pitaya cutting rooting, to further optimize the technical system of commercial pitaya seedling cultivation.

## 5. Conclusions

The results of this test showed that three auxin regulators, 1-naphthaleneacetic acid (NAA), potassium indole-3-butyrate (K-IBA) and indole-3-acetic acid (IAA), could significantly promote the root growth of ‘Jindu No.1’ pitaya cuttings, with a rooting rate of 100%, and all root indexes were significantly improved compared with the clean water control (*p* < 0.05). Among them, K-IBA had the best promoting effect on total root length and root number, NAA had the most significant improving effect on root thickness and root volume, while the regulatory effect of IAA series was unstable across experiments, and is not recommended for priority use in large-scale production.

The PCA results of the two rounds of primary and re-screening tests were highly consistent, which indicated that the root morphology of pitaya was regulated by two independent dimensions of “total growth–radial thickening”, and this regulatory mechanism had good stability and universality. The established three-level screening system of “broad-spectrum primary screening→gradient re-screening→soil culture scenario verification” can effectively eliminate the contingency of single-round tests and significantly improve the reliability of PGRs screening results.

The results of the soil culture verification test showed that the two optimal schemes of 125 mg·L^−1^ K-IBA and 400 mg·L^−1^ NAA still maintained the same regulatory characteristics and stable root-promoting effects as the hydroponic test under substrate cultivation conditions, and were not affected by cultivation methods. This study can provide technical reference for the large-scale, high-quality seedling cultivation of ‘Jindu No.1’ pitaya.

## Figures and Tables

**Table 1:** Summary of plant growth regulator (PGR) treatments across all experimental stages.

Experimental Stage	Treatment Code	PGR Reagent	Concentration (mg·L^−1^)
Primary Screening	CK	Clean water	-
	H1	14-hydroxylated brassinosteroid	0.075
	H2	24-epibrassinolide + 28-epibrassinolide mixture	0.05
	H3	28-epibrassinolide	0.04
	H4	28-homobrassinolide	0.07
	H5	NAA	200
	H6	IAA	250
	H7	IBA	200
	H8	K-IBA	250
	H9	S-ABA	4
	H10	GA_3_	150
	H11	6-BA	30
	H12	Sodium nitrophenolate	9
	H13	CPPU	20
	H14	DA-6	25
Re-Screening	CK	Clean water	-
	H5-1	NAA	800
	H5-2	NAA	400
	H5-3	NAA	200
	H5-4	NAA	100
	H5-5	NAA	50
	H6-1	IAA	1000
	H6-2	IAA	500
	H6-3	IAA	250
	H6-4	IAA	125
	H6-5	IAA	62.5
	H8-1	K-IBA	1000
	H8-2	K-IBA	500
	H8-3	K-IBA	250
	H8-4	K-IBA	125
	H8-5	K-IBA	62.5
Soil Culture Verification	CK	Clean water	-
	T1	K-IBA	125
	T2	NAA	400

**Table 2:** Sources and specifications of PGR reagents used in this study.

PGR Reagent	Abbreviation	Manufacturer	Purity/Content
14-hydroxylated brassinosteroid	-	Chengdu New Sun Crop Science Co., Ltd., Chengdu, China	0.0075% (*v*/*v*)
24-epibrassinolide + 28-epibrassinolide mixture	-	Qingdao Haina Biotechnology Co., Ltd., Qingdao, China	0.0075% (*v*/*v*)
28-epibrassinolide	-	Apuri (Jiaozuo) Chemical Co., Ltd., Jiaozuo, China	0.01% (*v*/*v*)
28-homobrassinolide	-	Jiangxi Xinbang Biochemical Co., Ltd., Nanchang, China	0.01% (*v*/*v*)
1-naphthaleneacetic acid	NAA	Sichuan Guoguang Agrochemical Co., Ltd., Chengdu, China	20% (*w*/*w*)
Indole-3-acetic acid	IAA	Fuzhou Feijing Biotechnology Co., Ltd., Fuzhou, China	99.9% (*w*/*w*)
Indole-3-butyric acid	IBA	Hubei Yipule Biological Technology Co., Ltd., Wuhan, China	1.2% (*v*/*v*)
Potassium indole-3-butyrate	K-IBA	Zhengzhou Yinzhihai Chemical Products Co., Ltd., Zhengzhou, China	98% (*w*/*w*)
S-abscisic acid	S-ABA	Sichuan Runer Technology Co., Ltd., Chengdu, China	0.1% (*v*/*v*)
Gibberellic acid	GA_3_	Sichuan Runer Technology Co., Ltd., Chengdu, China	3% (*v*/*v*)
6-benzylaminopurine	6-BA	Sichuan Runer Technology Co., Ltd., Chengdu, China	2% (*v*/*v*)
Sodium nitrophenolate	-	Shenzhen Noposion Agrochemicals Co., Ltd., Shenzhen, China	5-nitroguaiacol sodium 0.3% (*v/v*); Sodium p-nitrophenolate 0.9% (*v*/*v*); Sodium o-nitrophenolate 0.6% (*v/v*)
Forchlorfenuron	CPPU	Sichuan Guoguang Agrochemical Co., Ltd., Chengdu, China	0.1% (*v*/*v*)
Diethyl aminoethyl hexanoate	DA-6	Zhengzhou Yinzhihai Chemical Products Co., Ltd., Zhengzhou, China	98% (*w*/*w*)

**Table 3:** Root morphological traits of pitaya under soil culture conditions.

Test Code	Average Root Number (Roots)	Average Root Length (cm)	Average Root Surface Area (cm^2^)	Average Root Diameter (mm)	Average Root Volume (cm^3^)
CK	14.4 ± 1.57 b	214.75 ± 30.62 b	45.00 ± 7.79 b	0.66 ± 0.02 b	0.75 ± 0.16 b
T1	26.4 ± 1.75 a	351.11 ± 22.4 a	71.37 ± 5.92 a	0.65 ± 0.02 b	1.16 ± 0.13 a
T2	24.2 ± 1.24 a	334.87 ± 14.42 a	65.9 ± 3.28 a	0.85 ± 0.01 a	1.26 ± 0.1 a

Note: Data in the table are mean ± standard error. For the data in the same column, values followed by the same letter indicate no significant difference, while different letters indicate significant difference (*p* < 0.05, Duncan’s new multiple range test). The letter “a” represents the group with the highest value in the corresponding column.

## Data Availability

The data presented in this study are available on request from the corresponding author. The data are not publicly available due to privacy restrictions.

## Supplementary Materials

The following supporting information can be downloaded at: https://www.mdpi.com/article/10.3390/plants15091357/s1, Table S1. Root morphological indicators of pitaya under different PGR treatments. Table S2. Eigenvalues, variance contribution rates and cumulative variance contribution rates of principal components in the primary screening experiment. Table S3. Loading matrix and indicator contribution rate of PC1 and PC2 in the primary screening experiment. Table S4. Principal component scores and comprehensive ranking of each treatment group in the primary screening experiment. Table S5. Root morphological indicators of pitaya under PGR treatments with different concentrations. Table S6. Eigenvalues, variance contribution rates and cumulative variance contribution rates of principal components in the re-screening experiment. Table S7. Loading matrix and indicator contribution rate of PC1 and PC2 in the re-screening experiment. Table S8. Principal component scores and comprehensive ranking of each treatment group in the re-screening experiment. Table S9. Comparison of the ranking of dominant treatment groups between the primary and re-screening experiments. Table S10. Comparison of core PCA parameters between the primary and re-screening experiments.
