# Supplementary material for: Screening of Plant Growth Regulators for Promoting Rooting of Pitaya Cuttings
Source: Plants (Basel). 2026 Apr 29;15(9):1357. doi: 10.3390/plants15091357 (PMC13165443; doi:10.3390/plants15091357)
Supplement: Supplementary file 1 [file plants-15-01357-s001.zip › plants-4266468-supplementary.pdf]

Supplementary Materials

Screening of Plant Growth Regulators for Promoting Rooting of Pitaya Cuttings

The following supplementary materials are provided to support the conclusions of the main text. All abbreviations in this document are consistent with those in the main text.

Table S1. Root morphological indicators of pitaya under different PGR treatments.

Test Code	Average Root Number (roots)	Average Root Length (cm)	Average Root Surface Area (cm^2)	Average Root Diameter (mm)	Average Root Volume (cm^3)
CK	3.17±0.54d	8.96±1.88f	2.06±0.5d	0.69±0.06c	0.04±0.01d
H1	3.78±0.83d	16.89±4.27ef	4.31±1.04d	0.74±0.02c	0.08±0.02d
H2	5.40±0.56cd	30.29±5.87cdef	6.88±1.44d	0.72±0.03c	0.13±0.03d
H3	4.50±0.54d	26.37±4.47def	6.73±1.26d	0.79±0.03bc	0.14±0.03d
H4	5.78±0.7cd	26.09±4.78def	6.18±1.07d	0.78±0.04bc	0.12±0.02d
H5	14.5±2.09abc	57.63±9.58c	19.36±3.16bc	1.11±0.07ab	0.53±0.09bc
H6	17.30±1.76ab	84.74±6.92b	26.11±3.33b	0.95±0.07abc	0.66±0.12b
H7	6.00±0.87cd	36.92±7.59cdef	8.77±1.82cd	0.76±0.03bc	0.17±0.04cd
H8	21.5±1.63a	124.67±10.51a	38.34±3.89a	0.97±0.04ab	0.95±0.12a
H9	5.50±0.96cd	36.01±6.19cdef	9.95±1.86cd	0.89±0.04ab	0.22±0.05cd
H10	3.33±0.82d	20.07±4.01def	4.99±1.08d	0.82±0.1abc	0.12±0.02d
H11	7.22±0.94cd	44.1±5.85cde	10.39±1.44cd	0.75±0.02c	0.23±0.03cd
H12	8.70±0.94bcd	53.55±7.61cd	11.93±1.8cd	0.73±0.03c	0.21±0.03cd
H13	1.67±0.33d	2.14±0.36f	0.73±0.17d	1.07±0.06a	0.02±0.01d
H14	6.50±0.69cd	36.89±9.61cdef	10.56±2.25cd	0.95±0.02abc	0.25±0.04cd

Note: Data in the table are mean ± standard error. For the data in the same column, values followed by the same letter indicate no significant difference, while different letters indicate significant difference ($P<0.05$, Duncan's new multiple range test). The letter "a" represents the group with the highest value in the corresponding column.

Table S2. Eigenvalues, variance contribution rates and cumulative variance contribution rates of principal components in the primary screening experiment

Principal Component	Eigenvalue	Variance Contribution Rate (%)	Cumulative Variance Contribution Rate (%)
PC1	4.5036	84.0665	84.0665
PC2	0.8126	15.1692	99.2357
PC3	0.0268	0.4997	99.7345
PC4	0.0142	0.2642	99.9996
PC5	0.0000	0.0004	100

Table S3. Loading matrix and indicator contribution rate of PC1 and PC2 in the primary screening experiment

Indicator	PC1		PC2	
	Loading Coefficient	Contribution Rate (%)	Loading Coefficient	Contribution Rate (%)
Average root number (roots)	1.0217	23.08	-0.0963	1.11
Average root length (cm)	1.0014	22.28	-0.2377	6.88
Average root surface area (cm2)	1.0265	23.29	-0.1234	1.85
Average root diameter (mm)	0.5835	7.64	0.8547	88.92
Average root volume (cm3)	1.0309	23.71	-0.0346	0.12
Total	--	100	--	100

Table S4. Principal component scores and comprehensive ranking of each treatment group in the primary screening experiment

Test Code	PC1 Score	PC2 Score	Comprehensive Score	Comprehensive Ranking
H8	5.5311	-0.5828	4.5601	1
H6	3.3143	-0.0710	2.7765	2
H5	2.3738	1.4562	2.2181	3
H14	0.0331	0.8446	0.1550	4
H9	-0.2793	0.4658	-0.1655	5

H12	-0.0285	-1.1048	-0.1909	6
H11	-0.3013	-0.6205	-0.3474	7
H7	-0.6410	-0.4360	-0.6053	8
H4	-1.0177	-0.1522	-0.8777	9
H3	-1.0390	-0.0707	-0.8847	10
H2	-1.0520	-0.6125	-0.9771	11
H13	-1.6365	2.2581	-1.0336	12
H1	-1.6067	-0.2747	-1.3922	13
H10	-1.5886	-0.5906	-1.4257	14
CK	-2.0618	-0.5088	-1.8097	15

Table S5. Root morphological indicators of pitaya under PGR treatments with different concentrations

Test Code	Average Root Number (roots)	Average Root Length (cm)	Average Root Surface Area (cm^2)	Average Root Diameter (mm)	Average Root Volume (cm^3)
CK	3.67±0.84e	12.69±4.26f	3.42±1.13e	0.83±0.05bc	0.07±0.02e
H8-1	22.00±1.93a	88.54±12.67ab	23.05±3.13ab	0.84±0.03bc	0.48±0.07abc
H8-2	14.83±3.91abcd	75.05±21.31abc	19.12±5.3abc	0.79±0.05bcd	0.39±0.11bcd
H8-3	19.83±2.02abc	107.98±21.16a	24.35±5.16ab	0.81±0.02bcd	0.44±0.1abc
H8-4	20.83±3.66ab	108.89±20.84a	27.33±6.36a	0.71±0.07bcde	0.57±0.16ab
H8-5	11.50±0.96de	75.46±6.28abc	19.25±1.6abc	0.81±0.02bcd	0.39±0.04bcd
H6-1	11.17±1.38de	36.96±5.41cdef	7.89±1.31de	0.67±0.03cde	0.14±0.03de
H6-2	9.17±0.91de	32.74±6.34def	6.26±1.22def	0.60±0.04e	0.11±0.02e
H6-3	9.67±1.43de	34.77±7.21def	7.15±1.44def	0.69±0.05cde	0.12±0.03e
H6-4	13.5±1.63bcd	62.29±12.28bcde	14.98±3.19bcd	0.76±0.02cde	0.29±0.07cde
H6-5	12.83±1.49cd	68.64±8.3abcd	15.44±2.17bcd	0.71±0.05bcde	0.28±0.05cde
H5-1	7.83±2.09de	11.88±3.6f	2.42±0.73e	0.65±0.02cde	0.04±0.01e
H5-2	21.50±6a	69.29±19.57abcd	23.77±6.18ab	1.10±0.08a	0.66±0.17a

H5-3	9.17±1.38de	29.63±7.69def	9.53±2.16cde	1.05±0.08a	0.25±0.06cde
H5-4	6.80±0.91de	26.26±2.27ef	7.32±1.08de	0.86±0.08b	0.17±0.03de
H5-5	8.50±1.98de	34.12±9.23def	11.23±3.23cde	1.03±0.04a	0.30±0.09cde

Note: Data in the table are mean ± standard error. For the data in the same column, values followed by the same letter indicate no significant difference, while different letters indicate significant difference (*P*<0.05, Duncan's new multiple range test). The letter "a" represents the group with the highest value in the corresponding column.

Table S6. Eigenvalues, variance contribution rates and cumulative variance contribution rates of principal components in the re-screening experiment

Principal Component	Eigenvalue	Variance Contribution Rate (%)	Cumulative Variance Contribution Rate (%)
PC1	4.0454	75.8514	75.8514
PC2	1.1296	21.1793	97.0308
PC3	0.1216	2.2792	99.3100
PC4	0.0365	0.6836	99.9936
PC5	0.0003	0.0064	100

Table S7. Loading matrix and indicator contribution rate of PC1 and PC2 in the re-screening experiment

Indicator	PC1		PC2	
	Loading Coefficient	Contribution Rate (%)	Loading Coefficient	Contribution Rate (%)
Average root number (roots)	0.9797	23.72	-0.1554	2.14
Average root length (cm)	0.9684	23.18	-0.2989	7.91
Average root surface area (cm2)	1.0259	26.02	-0.0558	0.28
Average root diameter (mm)	0.3003	2.23	0.9859	86.05
Average root volume (cm3)	1.0026	24.85	-0.2023	3.62
Total	--	100	--	100

Table S8. Principal component scores and comprehensive ranking of each treatment group in the re-screening experiment

Test Code	PC1 Score	PC2 Score	Comprehensive Score	Comprehensive Ranking
H5-2	3.0004	1.8906	2.6602	1
H8-4	3.1182	-1.1729	2.1182	2
H8-1	2.5131	-0.2215	1.8499	3
H8-3	2.5442	-0.6592	1.7981	4
H8-2	1.1117	-0.2679	0.7905	5
H8-5	0.8554	-0.0467	0.6538	6
H6-4	0.1977	-0.4173	0.0626	7
H6-5	0.2105	-0.7639	0.0034	8
H5-5	-0.6239	1.7852	-0.0896	9
H5-3	-0.8473	1.9138	-0.2354	10
H5-4	-1.6908	0.7071	-1.1289	11
H6-1	-1.3855	-0.7972	-1.2269	12
H6-3	-1.6268	-0.6488	-1.3748	13
H6-2	-1.9147	-1.1961	-1.7106	14
CK	-2.7355	0.5939	-1.9435	15
H5-1	-2.7266	-0.6988	-2.2274	16

Table S9. Comparison of the ranking of dominant treatment groups between the primary and re-screening experiments

Experiment Round	Test Code	Average Root Number (roots)	Average Root Length (cm)	Average Root Surface Area (cm^2)	Average Root Diameter (mm)	Average Root Volume (cm^3)	Comprehensive Ranking
Primary screening	CK	3.17±0.54	8.96±1.88	2.06±0.5	0.69±0.06	0.04±0.01	15
	H5	14.5±2.09	57.63±9.58	19.36±3.16	1.11±0.07	0.53±0.09	3
	H6	17.3±1.76	84.74±6.92	26.11±3.33	0.95±0.07	0.66±0.12	2
	H8	21.5±1.63	124.67±10.51	38.34±3.89	0.97±0.04	0.95±0.12	1

Re-screening	CK	3.67±0.84	12.69±4.26	3.42±1.13	0.83±0.05	0.07±0.02	15
	H5-2	21.5±6	69.29±19.57	23.77±6.18	1.1±0.08	0.66±0.17	1
	H6-4	13.5±1.63	62.29±12.28	14.98±3.19	0.76±0.02	0.29±0.07	7
	H8-4	20.83±3.66	108.89±20.84	27.33±6.36	0.7±0.07	0.57±0.16	2

Note: Data in the table are mean ± standard error.

Table S10. Comparison of core PCA parameters between the primary and re-screening experiments

PCA Parameter	Primary Screening Experiment	Re-screening Experiment	Difference Range	Consistency Judgment
Cumulative variance contribution rate of the first two principal components	99.24%	97.03%	2.21%	Highly consistent
Variance contribution rate of PC1	84.07%	75.85%	8.22%	Consistent
Variance contribution rate of PC2	15.17%	21.18%	6.01%	Consistent
High-contribution indicator cluster of PC1	Average root number, length, surface area, volume	Average root number, length, surface area, volume	--	Completely consistent
Total contribution rate of high-contribution indicators of PC1	92.36%	97.78%	5.42%	Highly consistent
Dominant indicator of PC2	Average root diameter (88.92%)	Average root diameter (86.05%)	2.87%	Highly consistent
